# Triterpenes and the Antimycobacterial Activity of *Duroia macrophylla* Huber (Rubiaceae)

**DOI:** 10.1155/2013/605831

**Published:** 2013-02-12

**Authors:** Daiane Martins, Lillian Lucas Carrion, Daniela Fernandes Ramos, Kahlil Schwanka Salomé, Pedro Eduardo Almeida da Silva, Andersson Barison, Cecilia Veronica Nunez

**Affiliations:** ^1^Bioprospection and Biotechnology Laboratory, National Research Institute of Amazonia (INPA), 69060-001 Manaus, AM, Brazil; ^2^Mycobacterial Laboratory, Federal University Foundation of Rio Grande (FURG), 96200-190 Rio Grande, RS, Brazil; ^3^NMR Laboratory, Department of Chemistry, Federal University of Paraná (UFPR), 81530-900 Curitiba, PR, Brazil

## Abstract

*Duroia macrophylla* popularly known as “cabeça-de-urubú,” “apuruí,” or “puruí-grande-da-mata” occurs in the Amazon Forest. Its leaves and branches were collected twice and extracted with dichloromethane and methanol. All extracts were subjected to phytochemical investigation and terpenes and flavonoids were found in all dichloromethane and methanol extracts, respectively. Methanol extracts from both branches (1st collection) and leaves (2nd collection) presented hydrolyzed tannins, yet alkaloids were only detected in the dichloromethane and methanol extracts from branches at the 2nd collection. Phenol compounds were found in both dichloromethane extracts' collections. The action of every extract was assayed against *Mycobacterium tuberculosis* (RMPr, H37Rv, and INHr strains), showing that the dichloromethane extract from leaves (1st collection) has the major biological activity, with a MIC of 6.25 **μ**g/mL for the INHr strain, 25.0 **μ**g/mL for the RMPr strain, and ≤6.25 **μ**g/mL for the H37Rv strain. The chromatographic fractioning of the dichloromethane extract from leaves (1st collection) yielded the isolation of two triterpenes: oleanolic and ursolic acids, which were identified by NMR analysis and reported for the first time in the *Duroia* genus.

## 1. Introduction

Rubiaceae is the largest family in the Magnoliopsida class, encompassing around 550 genera and 9,000 species being used in several ethnomedicinal practices [1]. The family is characterized by the production of several classes of secondary metabolites with a great pharmacological potential, mainly alkaloids, terpenes, quinovic acid glycosides, flavonoids, and coumarins with antibacterial properties [2]. Rubiaceae plants' secondary metabolites have been investigated scientifically for antimicrobial activities and a large number of plant products have shown to inhibit the growth of pathogenic microorganisms [3–6]. A literature review article pertaining to Rubiaceae species reveals that 48 out of 611 genera showed a wide range of antibacterial [3] and antimycobacterial activities [4].

The *Duroia* genus, belonging to Gardenieae tribe and Ixoroideae subfamily, holds about 30 species but few studies have been carried out on this genus. *D. hirsuta*, which is used as folk healing medicine, is one of these species which has undergone investigation and showed antimycobacterial activity against *Mycobacterium phlei* [7] and antiviral activity against *Herpes simplex viruses* (HSV) on *in vitro* studies [8]. One flavone, one lactone iridoid, one flavonol [9] and one tetracyclic iridoid [10] were isolated from its root petroleum ether and CHCl_3_ extract. There is still a large number of species with no chemical or biological study.

*Duroia macrophylla* Huber, popularly known as “cabeça-de-urubú,” “apuruí,” or “puruí-grande-da-mata,” occurs in the Amazon Forest [11]. To the best of our knowledge, no chemical or biological investigations other than ours [12, 13] have been carried out on this species as yet. Hence this work aims to evaluate the antimycobacterial activity of their extracts and isolate and identify the substances present in *D. macrophylla* active extracts. 

## 2. Material and Methods

### 2.1. Plant Material

Two collections were performed, the first one, at the “A. Ducke” Forest Reserve, 26 km from Manaus, was carried out on December 5th, 2008, and a voucher specimen (222383) was deposited at the Herbarium of the Botanical Research Coordination of the National Research Institute of Amazonia (INPA). The second one at the Natural Heritage Private Reserve, locally known as “Cachoeira da Onça,” in “Presidente Figueiredo” County, AM, was carried out on May 18th, 2011. A voucher specimen (222501) was deposited at the same Herbarium. 

### 2.2. Extracts Preparation

Plant material (leaves and branches) was dried in an oven at 50°C and powdered. Each plant part was extracted three times separately, first with dichloromethane (DCM) followed by methanol (MeOH), in a sonic bath for 20 minutes. After filtration, DCM and MeOH extracts were concentrated under reduced pressure.

### 2.3. Phytochemical Investigation

The extracts were analyzed following the methodology described by Matos [14], as well as by thin layer chromatography (TLC) (Merck) using silica with UV_254_ fluorescence detector on aluminum support, eluted with appropriated systems, and revealed with UV light (*λ* = 254 and 365 nm), sulfuric *p*-anisaldehyde, Ce(SO_4_)_2_,   2,2-diphenyl-1-picrylhydrazyl (DPPH), FeCl_3_, and Dragendorff. Chemical extract profile was identified through ^1^H-NMR on an Anasazi NMR spectrometer operating at 1.4 Tesla (60 MHz). 

### 2.4. Extract Fractionation

**Figure 1:**
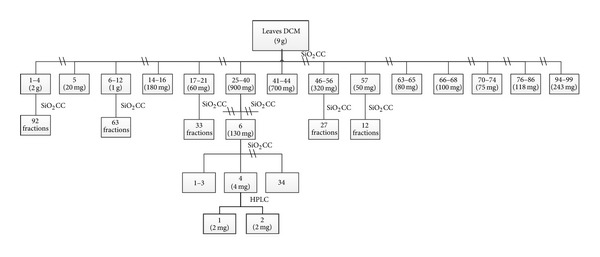
Schematic representation of* D. macrophylla* dichloromethane extract of leaves (1st collection) fractionation. DCM: dichloromethane; CC: chromatographic column; HPLC: high-performance liquid chromatography.

Dichloromethane extract from leaves (1st collection) (9 g) was submitted to a chromatographic column (CC) fractionation on silica gel (332 g), eluted with gradients of hexane/ethyl acetate and ethyl acetate/methanol, yielding 99 fractions with 50 mL each. Fraction 25–40 (900 mg) was fractionated on silica gel (90 g) CC and eluted with hexane/ethyl acetate and ethyl acetate/methanol gradients, yielding 42 fractions with 20 mL each. Fraction 25–40.6 (130 mg) was fractionated on silica gel (17 g) CC and eluted with hexane/ethyl acetate and ethyl acetate/methanol gradients, yielding 19 fractions with 10 mL each. Afterwards, fraction 25–40.6.4 (4 mg) was submitted to high-performance liquid chromatography (HPLC) analysis. HPLC was performed with a Shimadzu system SCL-10AVP, processing software programs CLASS VP, dual LC-6AD pumps, 10AF autosampler, SPD-M20 diode-array detector, cyanopropyl column (250 × 10 mm, 4 *μ*m particle sizes, Luna-Phenomenex), with acetonitrile : water (90 : 10) as the isocratic mobile phase, at a 5 mL/min flow rate. The injection volume was 35 *μ*L. The resolved peaks retention times were 11.5 and 12 min, identified by NMR analyses as oleanolic acid (**1**) and ursolic acid (**2**), respectively (Figure 1).

All fractions were evaluated by TLC analysis, eluted with appropriated systems, and revealed under UV light exposure (*λ* = 254 and 365 nm), sulfuric *p*-anisaldehyde, Ce (SO_4_)_2_,   2,2-diphenyl-1-picrylhydrazyl (DPPH), FeCl_3_, and Dragendorff reagents. 

### 2.5. NMR Data

The NMR data was obtained at 295 K on a Bruker AVANCE 400 NMR spectrometer operating at 9.4 Tesla, observing ^1^H and ^13^C at 400 and 100 MHz, respectively. The spectrometer was equipped with a 5 mm multinuclear direct detection probe, with z-gradient. One-bond (HSQC) and long-range (HMBC) ^1^H-^13^C NMR correlation experiments were optimized for coupling constants ^1^ *J*_H,C_ and ^LR^ *J*_H,C_ of 140 and 8 Hz, respectively. All NMR chemical shifts were expressed in ppm related to TMS signal at 0.00 ppm as internal reference, and samples were dissolved in CDCl_3_. 

### 2.6. Antimycobacterial Activity

Resazurin microtiter assay (REMA) was used to evaluate the antimycobacterial activity. This method uses resazurin as an oxidoreduction indicator to evaluate the bacterial viability and contamination, in addition to analyzing the antimicrobial activity [15].

#### 2.6.1. Microorganisms

The extracts activity was evaluated against three *Mycobacterium tuberculosis* strains: one pan-sensible (H37Rv, ATCC 27294), one isoniazid monoresistant (INH, ATCC 35822) with mutation in *kat*G, codon S315T (AGC-ACC), and other rifampicin monoresistant (RMP, ATCC 35338), with mutation in *rpo*B, codon H526T (CAC-TAC). The strains were cultivated in Ogawa-Kudoh's medium at 37°C for nearly 14 days. The bacterial suspension of each strain was prepared in a sterile tube with glass pearls and turbidity adjusted with distillated water, according to Mc Farland scale's number 1 tube, which corresponds to approximately 3 × 10^8^ CFU/mL. Then, Middlebrook 7H9 medium was added to bacterial suspension in 1 : 20 ratio [15].

#### 2.6.2. Assay Procedure

Samples were first evaluated in 96-well microplates at a 200 *μ*g/mL concentration against the three *M. tuberculosis* strains. The assay started adding 75 *μ*L of Middlebrook 7H9 medium enriched with 10% of OADC (oleic acid, albumin, dextrose, and catalase) for* M. tuberculosis*, 75 *μ*L of each extract, and 75 *μ*L of inoculum. Then, 200 *μ*L of sterile water were added to each peripheral well, so as to avoid medium liquid evaporation when heater-incubated. Finally, the plate was incubated at 37°C for seven days.

#### 2.6.3. Minimum Inhibitory Concentration Determination

The extracts presenting an antimycobacterial activity at the 200 *μ*g/mL concentration screening were chosen to evaluate their minimum inhibitory concentration (MIC) value [16]. This value was determined by adding 100 *μ*L of medium, 100 *μ*L of extract (starting at 200 *μ*g/mL concentration on the first well and performing a 1 : 2 microdilution), and 100 *μ*L of bacterial inoculum in each well. Also, 200 *μ*L of sterile water was added to each peripheral well, in order to avoid medium liquid evaporation when heater-incubated. Then, the plate was incubated at 37°C for seven days.

#### 2.6.4. Bacterial Viability

Following the incubation period, 30 *μ*L of resazurin (0.02%) was added in each well and incubated for two days at 37°C. The biological activity was based on the color change, from blue to pink when an oxidoreduction reaction of the reagent occurs due to bacterial growth [15].

## 3. Results and Discussion

### 3.1. Phytochemical Investigation

All *Duroia macrophylla *extracts were analyzed in order to evaluate the chemical profile [17, 18]. Dichloromethane extracts from branches and leaves in both collections showed to be rich in terpenes. Regarding methanolic extracts, only those from branches (1st collection) and leaves (2nd collection) showed the presence of both terpenes and hydrolyzed tannins. Alkaloids were only detected on dichloromethane and methanolic extracts from branches (2nd collection). All methanolic extracts showed the presence of flavonoids. All dichloromethane extracts from branches showed the presence of phenolic compounds. ^1^H-NMR spectra analysis showed the presence of aromatic substances in the methanolic extract of branches (1st collection), with several signals between 6.50 and 7.80 ppm. 

### 3.2. Substances Isolation and Identification

Following crude extracts chemical and biological analysis, the dichloromethane extract from leaves (1st collection) was chosen to be fractionated, since it showed to be the most active against the three *Mycobacterium tuberculosis* strains (RMPr, H37Rv and INHr) (Table 2).

 Fraction 25–40.6 ^1^H-NMR data showed the presence of several signals in the shielded region between *δ*_H_ 0.7 and 1.2 (s), characteristic of methyl hydrogens; two signals at *δ*_H_ 5.31 (dd, *J* = 3.6; 3.5 Hz) and 5.27 (dd, *J* = 3.6; 3.5 Hz) characteristic of olefinic hydrogen, and also two signals at 3.23 (dd, *J* = 10.7; 4.7 Hz) and 3.22 (dd, *J* = 10.8; 4.9 Hz) which agree with carbinolic hydrogens. All this data suggests the mixture of two triterpenes. 

 HPLC fractionation of this mixture was performed in order to isolate them, and yielded two fractions, **1** and **2**, with retention times of 11.5 and 12.0 min. The ^1^H-NMR spectrum from fraction 25–40.6.4.1 showed the signal at *δ*_H_ 5.31 (dd, *J* = 3.6; 3.5 Hz) and from fraction 25–40.6.4.2, the signal at *δ*_H_ 5.27 (dd, *J* = 3.6; 3.5 Hz).

The substance **1** ^1^H-^13^C NMR (HSQC) correlation map showed the hydrogen at 5.31 ppm with the carbon at 122.8, which were identified as the vinilic C-12 carbon of oleanolic acid [7, 19] (Table 1). The signal in *δ*_C_ 180.0 was assigned to the carboxyl group (C-28). 

The ^1^H-NMR spectrum from fraction 25–40.6.4.2 showed several signals at the shielded region, between *δ*_H_ 0.79 and *δ*_H_ 1.72 characteristic of methyl hydrogens, moreover two signals were observed at 3.22 (dd, *J* = 10.8 and 4.9 Hz) and *δ*_H_ 5.27 (dd, *J* = 3.6 and 3.5 Hz) characteristic of olefinic hydrogen, which were assigned to H-12 in triterpenes, characterizing the ursanic skeleton of substance **2**.

When analyzing the ^13^C-NMR spectral data one can find seven methyl carbons (CH_3_), nine methylene carbons (CH_2_), seven methine carbons (CH) and seven non-hydrogenated carbons (C), resulting in thirty carbons characteristic of pentacyclic triterpenes. *δ*_C_ 179.6 from the carboxylic acid carbon (not hydrogenated), *δ*_C_ 137.9 characteristic of unhydrogenated olefinic carbon (sp^2^) and *δ*_C_ 125.9 of hydrogenated olefinic carbon are the major signals characteristic of a ursanic skeleton. These signals represent, carbons C-28, C-13 and C-12 in ursolic acid triterpene, respectively. 

On the other hand, the ^1^H-^13^C NMR (HSQC) correlation map showed correlation of the hydrogen at 5.27 ppm with the carbon at 125.9, which were identified as the vinilic C-12 carbon and the multiplicity of the signals corresponding to H-18 and related CH_3_-29 and CH_3_-30 determined the ursolic acid.

 In the two-dimensional ^1^H-^1^H NMR (COSY) correlation map, the following correlations are observed: hydrogen H-11 (*δ* 1.91) with H-12 (*δ* 5.27). 

It is common to isolate the ursolic acid with oleanolic acid mixture due to molecule similarity, yet a few differences between them enable telling them apart through NMR, due to the difference between the H-18, C-18, C-12, C-13 and C-29 [20] chemical shifts, and mainly on account of H-29 being a doublet for ursolic acid, and a singlet for oleanolic acid. 

**Figure 2:**
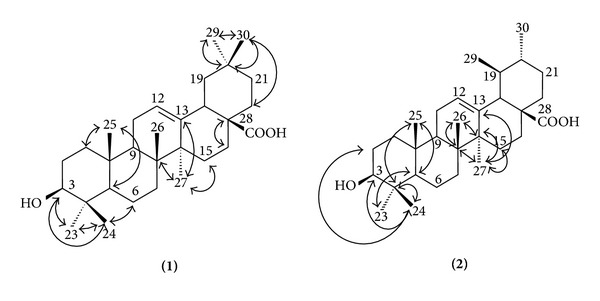
Structures of oleanolic and ursolic acids and their ^1^H-^13^C long-range correlations.

^1^H-NMR spectra and HSQC and HMBC NMR correlation maps overall analysis as well as comparison with literature data [7] enabled the complete structure to be determined as the triterpenes oleanoic and ursolic acids (Table 1) (Figure 2). 

The mass spectra analysis of each triterpene isolated showed the molecular ion peak at *m*/*z* = 456 u, and showed the common fragmentation pattern of triterpenes, described in the literature [21]. All these data together confirmed to be the triterpenes, ursolic acid and oleanolic acid. To the best of our knowledge, this is the first report of these two triterpenes in *Duroia* genus.

### 3.3. Antimycobacterial Activity

All extracts showed activity against *M. tuberculosis* at least for one strain, except for the methanol extract of branches (1st collection) (Table 2). The dichloromethane extract of leaves (1st collection) showed the highest activity, with a MIC of 6.25 *μ*g/mL for INHr strain, 25.0 *μ*g/mL for RMPr strain and ≤6.25 *μ*g/mL for H37Rv strain. Triterpenes oleanoic and ursolic acids were isolated from this extract. The methanolic extract of leaves (2nd collection) that showed a MIC of 12.5 *μ*g/mL for INHr strain was the second most active one (Table 2). 

 The wide variety of natural products chemical structures plays a major role on the development of new antimycobacterial drugs generations, as shown in the extensive literature revision made by Copp [22], from 1990 to 2002, which uncovered 352 substances isolated from natural products (terrestrial and marine) presenting an antimycobacterial activity and a MIC ≤ 64 *μ*g/mL.

The highest activity of the dichloromethane extract from leaves (1st collection) in this work could be attributed to the presence of terpenes. Several studies, such as those performed by Newton et al. [23], Cantrell et al. [24], Copp [22], Seidel and Taylor [25], Aguiar et al. [26], and Higuchi et al. [27], showed terpenes to be responsible for the antimycobacterial activity. 

Extracts and compounds from other Rubiaceae species, such as *Duroia hirsuta* and *Psychotria vellosiana*, showed activity against *Mycobacterium phlei *[6] and *M. tuberculosis *and *M. kansasii*, respectively [28]. According to some authors, the antimycobacterial activity can also be related to the presence of alkaloids, normally found in Rubiaceae species [29, 30]. 

Out of the 27 assayed fractions present in this work, only fraction 63-65 was as active against *M. tuberculosis* INHr strain as the dichloromethane extract of leaves (1st collection) (MIC of 25 *μ*g/mL). Among the others, 15 fractions were active against *M. tuberculosis* H37Rv strain (MIC between 50 and 200 *μ*g/mL), 17 fractions were active against INHr strain (MIC between 25 and 200 *μ*g/mL), and 16 fractions were active against RMPr strain (MIC between 50 and 200 *μ*g/mL) (Table 3). Fraction 25-40.6 was active against the three strains, with a MIC of 200 *μ*g/mL and their fractionation yielded the substances **1** and **2** corresponding to the triterpenes oleanoic and ursolic acids, respectively. 

Studies conducted by Higuchi et al. [27] reported the oleanolic and ursolic acids' mixture MIC to be 62.5 *μ*g/mL. Other studies showed the growth inhibitory activity against *Mycobacterium tuberculosis *with a MIC of 16 *μ*g/mL for oleanolic acid and 50 *μ*g/mL for ursolic acid [24]. 

Ge et al. [31] also obtained a better MIC value for oleanolic acid (MIC of 28.7 *μ*g/mL) than that for ursolic acid (MIC of 41.9 *μ*g/mL). These latter examples showed each isolated compound to exhibit a better MIC than the mixture's. 

The high lipophilicity of terpenes is probably the main factor that allows their penetration through the mycobacterial cell wall [27].

Other studies showed these substances inhibited 99% the growth of *M. tuberculosis* H37Rv [32]. The literature data reported that oleanolic acid has a synergistic effect when combined with isoniazid, rifampicin, or ethambutol (first line antitubercular drugs) [32]. 

According to Pauli et al. [33], a crude extract MIC may or not be a reliable antimycobacterial activity indicator since such extracts could hold active compound antagonist substances decreasing the MIC. Otherwise, a crude extract could hold compound agonists with increasing effects on MIC, the so called synergism effect. According to the author an extract with high activity (lower MIC) could present large amounts of compounds with moderated antimycobacterial activity. In other scenario, crude extracts with moderated MIC could hold small amounts of chemically active compounds. When the extract loses its activity during the fractionation, it could be due to a synergism effect between the substances on it. Therefore, the combined action of two or more substances can result on a biological effect higher than any single one's. Hence, more thorough studies are necessary to find which substances should be mixed in order to attain the desirable antimycobacterial activity.

## Figures and Tables

**Table 1:** ^
1^H and ^13^C NMR chemical shifts (*δ*, ppm) data of oleanolic and ursolic acids (400 MHz, CDCl_3_).

Position	Oleanolic acid			Ursolic acid		
	*δ*_C_	*δ*_H_ (multiplicity)	*δ*_C_ Literature [7]	*δ*_C_	*δ*_H_ (multiplicity)	*δ*_C_ Literature [7]
1	38.5	1.63 (m)	39.0	38.6	1.72 (m)	39.2
2	28.1	1.60 (m)	28.1	28.2	1.60 (m)	28.2
3	79.1	3.23 (dd; *J* = 10.7; 4.7 Hz)	78.2	78.7	3.22 (dd; *J* = 10.8; 4.9 Hz)	78.2
4	38.8	—	39.4	38.5	—	39.6
5	55.3	0.74 (m)	55.9	55.2	1.34 (m)	55.9
6	18.8	1.54 (m)	18.8	18.3	1.60 (m)	18.8
7	32.7	1.49 (m)	33.4	32.9	1.72 (m)	33.7
8	39.3	—	39.8	39.5	—	40.1
9	47.6	1.54 (m)	48.2	47.3	1.60 (m)	48.1
10	37.0	—	37.4	37.0	—	37.5
11	23.8	0.94 (m)	23.8	23.7	1.91 (m)	23.7
12	122.8	5.31 (dd; *J* = 3.6; 3.5 Hz)	122.6	125.9	5.27 (dd; *J* = 3.6; 3.5 Hz)	125.7
13	143.5	—	144.8	137.9	—	139.3
14	41.5	—	42.2	42.0	—	42.6
15	27.7	1.60 (m)	28.4	28.1	1.60 (m)	28.8
16	23.7	0.94 (m)	23.8	25.0	1.34 (m)	25.0
17	46.7	—	46.7	48.1	—	48.1
18	42.1	2.82 (m)	42.1	53.8	2.2 (m)	53.6
19	46.0	2.87 (m)	46.6	38.5	1.00 (m)	39.5
20	31.0	—	31.0	38.5	0.95 (m)	39.4
21	33.9	1.62 (m)	34.3	30.3	1.27 (m)	31.1
22	33.2	1.30 (m)	33.2	37.4	1.72 (m)	37.4
23	28.0	1.00 (s)	28.8	28.9	1.00 (s)	28.8
24	16.8	0.79 (s)	16.5	15.6	0.79 (s)	16.5
25	15.3	0.93 (s)	15.6	15.4	0.94 (s)	15.7
26	17.1	0.79 (s)	17.5	17.1	0.82 (s)	17.5
27	26.0	1.16 (s)	26.2	23.5	1.10 (s)	24.0
28	180.0	—	180.0	179.6	—	179.7
29	33.1	0.92 (s)	33.4	17.0	0.87 (d; *J* = 6.4 Hz)	17.5
30	23.7	0.94 (s)	23.8	21.4	0.97 (d; *J* = 6.3 Hz)	21.4

**Table 2:** Minimum inhibitory concentration (MIC) of *D. macrophylla* extracts against *M. tuberculosis* strains.

	*M. tuberculosis *					
Extracts	H37Rv		INHr		RMPr	
	(μg/mL)		(*μ*g/mL)		(*μ*g/mL)	
1st Collection						
Leaves DCM	S	6.25	S	25	S	≤ 6.25
Leaves MeOH	R	>200	R	>200	S	200
Branches DCM	S	100	S	100	S	100
Branches MeOH	R	>200	R	>200	R	>200

2nd Collection						
Leaves DCM	S	200	S	50	R	>200
Leaves MeOH	S	100	S	12.5	S	100
Branches DCM	S	25	S	50	R	>200
Branches MeOH	S	100	S	100	S	100

DCM: dichloromethane, MeOH: methanol, R: resistant, S: sensible, H37Rv: sensible strain, INHr: isoniazid resistant strain, RMPr: rifampicin resistant strain. Extract with MIC > 200 *µ*g/mL were considered inactive.

**Table 3:** Minimum inhibitory concentration (MIC) of dichloromethane fractions from the leaves of *D. macrophylla* (1st collection) against *M. tuberculosis *strains.

Fraction	*M. tuberculosis *					
	H37Rv (*μ*g/mL)		INHr (*μ*g/mL)		RMPr (*μ*g/mL)	
Fr 1–4	R	>200	R	>200	R	>200
Fr 1–4.17–20	R	>200	R	>200	R	>200
Fr 5	R	>200	R	>200	R	>200
Fr 6–12	S	50	S	100	S	100
Fr 6–12.30	R	>200	R	>200	R	>200
Fr 6–12.33–35	R	>200	R	>200	R	>200
Fr 6–12.38–63	R	>200	R	>200	R	>200
Fr14–16	S	100	S	50	S	100
Fr 17–21	R	>200	R	>200	R	>200
Fr 17.21.1–5	R	>200	R	>200	R	>200
Fr 25–40	S	200	S	200	S	200
Fr 25–40.2	R	>200	S	200	S	200
Fr 25–40.6	S	200	S	200	S	200
Fr 25–40.6.32	S	100	S	100	S	100
Fr 41–44	S	100	S	50	S	100
Fr 46–56	S	200	S	200	S	100
Fr 46–56.5	S	200	S	200	S	200
Fr 46–56.8–10	S	50	S	50	S	50
Fr 46–56.13–17	R	>200	R	>200	R	>200
Fr 57	S	100	S	200	S	200
Fr 57.6–12	S	100	R	>200	R	>200
Fr 63–65	S	100	S	25	S	100
Fr 66–68	S	200	S	100	S	200
Fr 70–74	R	>200	S	200	R	>200
Fr 76–86	R	>200	S	200	S	200
Fr 87–92	S	200	S	50	S	100
Fr 94–99	S	200	S	200	S	100

Fr: fraction, R: resistant, S: sensible, H37Rv: sensible strain, INHr: isoniazid resistant strain, RMPr: rifampicin resistant strain. Fractions with MIC > 200 *μ*g/mL were considered inactive.
